# Orthopaedic surgical documentation: pre-templated operation notes significantly improve documentation of surgical procedures

**DOI:** 10.1186/s13018-022-03484-9

**Published:** 2023-01-17

**Authors:** Fraser Sneddon, N. Fritsch, S. Mackenzie, D. Skipsey, I. A. Rankin

**Affiliations:** 1 Department of Trauma and Orthopaedics, NHS Highland, Inverness, Scotland; 2 Department of Trauma and Orthopaedics, NHS Tayside, Dundee, Scotland; 3 Department of Trauma and Orthopaedics, NHS Grampian, Aberdeen, Scotland

## Abstract

**Objectives:**

The Royal College of Surgeons of England (RCS) Good Surgical Practice guidance identifies essential criteria for surgical operation note documentation. The current quality improvement project aims to identify if using pre-templated operation notes for documenting fractured neck of femur surgery results in improved documentation when compared to freehand orthopaedic operation notes.

**Methods:**

A total of fourteen categories were identified from the RCS guidance as required across all the operations identified in this study. All operations for the month of October 2021 were identified and the operation notes analysed. Pre-templated operation notes were compared to freehand operation notes.

**Results:**

Ninety-seven cases were identified, of which 74 were freehand operation notes and 23 were pre-templated fractured neck of femur operation notes. All fourteen categories were completed in 13 (57%) of the templated operation notes versus 0 (0%) in the freehand operation notes (odds ratio 0.0052, 95% CI 0.0003 to 0.0945, *p* < 0.001). The median total number of completed categories was significantly higher in the templated op-note group compared to the freehand op-note group (templated median 14, range 12–14, vs. freehand median 11, range 9 to 13, *p* < 0.001). Logistic regression analysis of operation notes written by consultants or trainees identified trainees as more likely to document the antibiotic prophylaxis given (*p* = 0.025).

**Conclusions:**

Use of pre-templated operation notes results in significantly improved documentation. Adoption of generic pre-templated operation notes to improve surgical documentation should be considered across all operations.

## Introduction

The importance of accurate, legible, and detailed operation notes cannot be understated for all practicing surgeons. This is essential for clear communication between the peri-operative and post-operative periods, contributing to improved patient care and safety. Furthermore, it serves as a beneficial tool for research, audit, and medicolegal purposes [1]. Despite this importance being well known, up to 45% of operative documentation are inadequately completed and would be considered indefensible medicolegally [2]. Thus, the General Medical Council (GMC) recommends that operative documentation is as accurate, comprehensive, and legible as possible as part of the Good Medical Practice Guidance [3].

The Royal College of Surgeons England (RCSEng) published guidelines for maintaining Good Surgical Practice in 2008 (updated in 2014) [4]. As part of this publication guidelines for the standardisation of intraoperative documentation were provided, which should accompany the patient post-operatively to enable safe and efficient continuity of care. These guidelines have provided a basis for undertaking audits on operative documentation. Recent publications from multiple institutions highlight that compliance with these guidelines remains inadequate [1, 5, 6].

Two types of handwritten operative documentation notes are used at our centre to record the intraoperative details of all orthopaedic trauma operations. The first operative documentation sheet is a freehand operation note used for most procedures. The alternative, a pre-templated note, is used for all operations performed in the treatment of a fractured neck of femur (NOF).

**Fig. 1:**
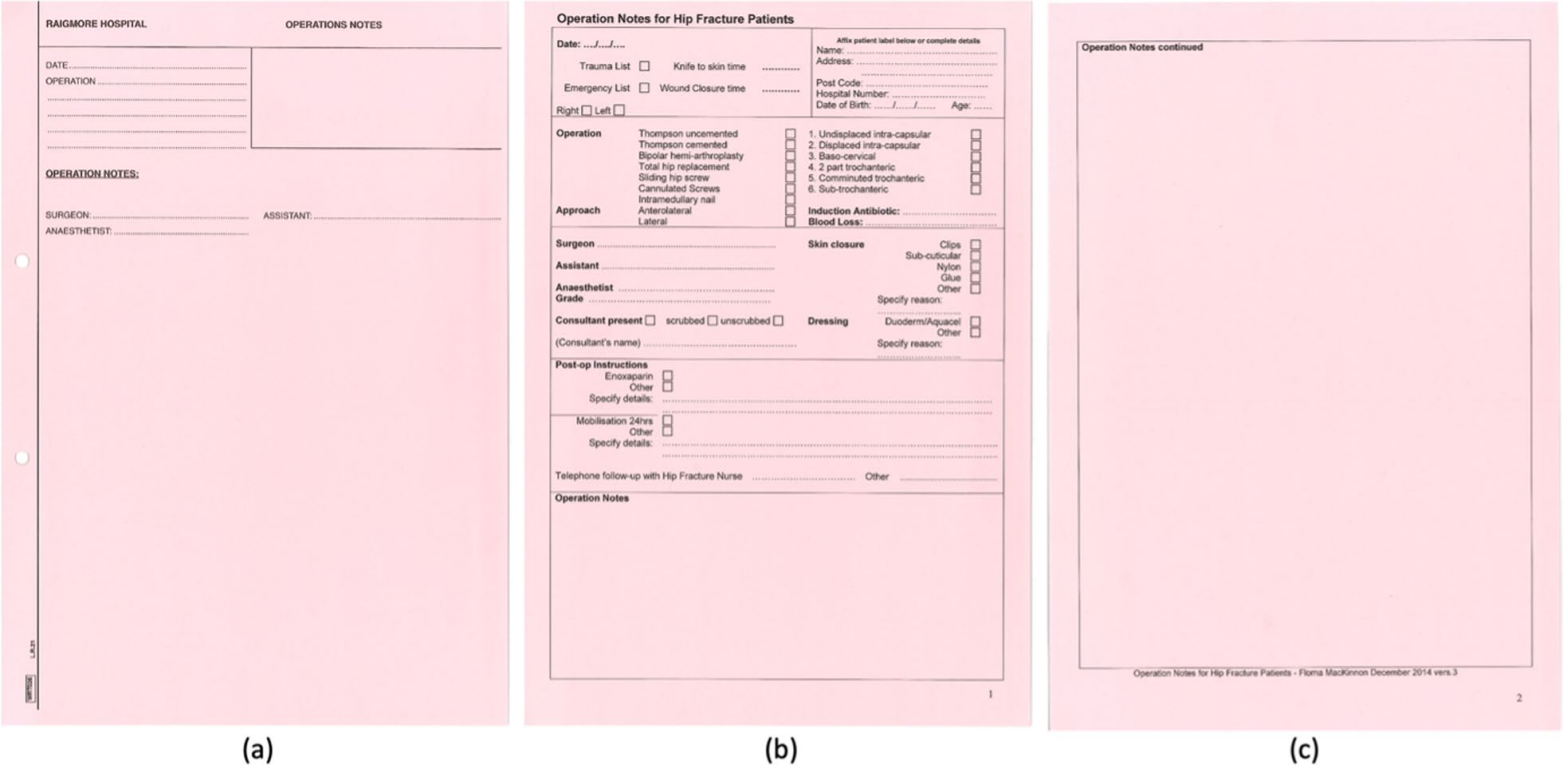
Raigmore Hospital Operation Proformas. **a** Standard freehand operation sheet **b** Front page pre-templated NOF Fracture operation sheet **c** Back Page pre-templated NOF Fracture operation sheet

The freehand operation note contains a space to affix a patient label and specific headings of date, operation, surgeon, assistant, and anaesthetist (Fig. 1a). The pre-templated NOF fracture operation sheet contains six separate sections as follows (Fig. 1b & c).

- Section One—space to affix patient label or enter details manually (name, address, postcode, hospital number, date of birth and age).
- Section Two—titles: date, knife to skin time and wound closure time. Check boxes: trauma / emergency list and right / left side.
- Section Three—titles: induction antibiotics and blood loss. Check boxes: operation, approach, and injury.
- Section Four—titles: surgeon, assistant, anaesthetist, grade. Check boxes: consultant present, skin closure and dressing.
- Section Five—space for post-operative Instructions, specifically regarding venous thromboembolism prophylaxis, mobilisation and follow up.
- Section Six—space for handwritten documentation of operation notes.

The aim of this study was to investigate if there was increased compliance with RCSEng guidelines for intraoperative documentation when a pre-templated operation note format was used. Furthermore, we aimed to identify, interpret, and implement methods to improve documentation of the peri- and post-operative period with the goal of improving patient safety, continuity of care, and medicolegal integrity for the operating surgeon.

## Methods

All orthopaedic trauma operations carried out for the month of October 2021 were identified and handwritten operation notes were analysed. Data were collected according to The RSCEng guidance for operation note documentation as required across all operations carried out [4]. A total of fourteen points were identified from the guidance for collection: date, time, elective or emergency procedure, name of the operating surgeon and assistant, name of the anaesthetist, operative procedure carried out, incision, operative diagnosis, operative findings, details of closure technique, anticipated blood loss, antibiotic prophylaxis, detailed post-operative care instructions, and a signature.

Data analysis was performed using IBM SPSS statistics version 25 (IBM, New York, USA). Pearson Chi-Square was used to assess for significant differences across individual categorical variables. Odds ratio was performed for odds of completing all fourteen categories. Mann–Whitney U test was used to test for significant differences between groups for the median number of completed categories on operation notes. Binary logistic regression analysis was used to assess for significant differences in documented categories between consultants and trainees.

## Results

Ninety-seven cases were identified, of which 74 were freehand operation notes and 23 were templated NOF fracture operation notes. Completion of all fourteen categories was significantly higher in the templated op-note group compared to the standard freehand op-note group (Table 1); all fourteen categories were completed in 13 (57%) of the templated operation notes versus 0 (0%) in the freehand operation notes (odds ratio 0.0052, 95% CI 0.0003 to 0.0945, *p* < 0.001). The median total number of completed categories was also significantly higher in the templated op-note group compared to the freehand op-note group (templated median 14, range 12–14, vs. freehand median 11, range 9 to 13, *p* < 0.001). Individual categories significantly different (*p* < 0.001) included documentation of time, emergency or elective procedure, and anticipated blood loss.

**Table 1:** Results of Compliance with RCSEng guidance for standardisation of intraoperative documentation

Category documented	Freehand op-note (*n* = 74)	Templated op-note (*n* = 23)
Date	72 (97%)	23 (100%)
Time	1 (1%)	20 (87%)*
Elective or emergency	0 (0%)	23 (100%)*
Name of the operating surgeon and assistant	70 (95%)	23 (100%)
Name of the anaesthetist	72(97%)	21 (91%)
Operative procedure carried out	74 (100%)	22 (96%)
Incision	64 (88%)	22 (96%)
Operative diagnosis	74 (100%)	23 (100%)
Operative findings	74 (100%)	23 (100%)
Details of closure technique	73 (99%)	23 (100%)
Anticipated blood loss	1 (1%)	20 (87%)*
Antibiotic prophylaxis	58 (78%)	22 (96%)
Detailed post-operative care instructions	74 (100%)	23 (100%)
Signature	71 (96%)	21(91%)
All categories	0 (0%)	13 (57%)*

*denotes statistically significant value, *p* < 0.001

Logistic regression analysis identified documentation of antibiotic prophylaxis as significantly different between consultant and trainee operations notes (*p* = 0.025). Subsequent odds ratio analysis identified that trainees were significantly more likely to document the prophylactic antibiotic given when compared to consultants (odds ratio 3.78, 95% CI 1.13 to 12.59).

## Discussion

The keeping of accurate, legible, and detailed medical records remains a key component of good medical practice recommended by both the GMC and RCSEng [3, 4]. Its importance is highlighted with the increasing number of medicolegal cases in recent years, where documentation around the peri-operative period is required to be accurate and legible to provide a clear record of events [7]. Furthermore, clear peri-operative documentation is essential for continuity of care between the peri- and post-operative periods where it should allow for clear communication of events and the ongoing management plan for all parties involved in patient care. Our study demonstrated that pre-templated operation sheets significantly improve post-operative documentation and compliance with RCSEng guidance.

Our centre utilises two types of operative documentation notes: a freehand (Fig. 1a) and a pre-templated note used for NOF fracture operations (Fig. 1b & c). Whilst the pre-templated note observed in our study focused on operations in the treatment of neck of femur fractures, areas of significantly reduced documentation points were not specific to these procedures. As such, surgical documentation could be improved across all orthopaedic operations where a pre-templated format is adopted, with the inclusion of the key RCSEng guidelines as highlighted within this study. This has previously been shown to be beneficial across other specialties and institutions [8, 9].

In addition to completion of key details, difficulties with accurate and legible documentation of operative procedures remain an issue at many institutions [1, 5, 6]. As part of the updates made to the RCSEng guidance in 2014, it was stated that operation notes should preferably be typed [4]. Whilst there were no incidences of illegible handwriting in the notes reviewed within this study, the use of typed notes can improve legibility and therefore contribute to improved communication and increased medicolegal integrity when compared with handwritten documentation. As such, our recommendation would be to incorporate a pre-templated operation note as part of a saved electronic template.

We acknowledge that this study is limited with regards to being retrospective, only including cases from a single-centre and pre-templated operation notes only being used for cases related to NOF Fractures. Although we would argue these results are significant enough to recommend the use of pre-templated operation notes, further research could focus on development of a general template for use when documenting all operations. This would allow a prospective study comparing pre-templated operation notes to freehand, without the limitations of this study.

## Conclusion

The use of a pre-templated surgical documentation proforma note significantly increases full compliance with RCSEng guidance for the documentation of surgical procedures. Compliance with individual aspects was significantly increased at our centre across documentation of time, emergency or elective procedure, and anticipated blood loss. Surgical documentation across all orthopaedic procedures could be improved where a pre-templated standard surgical documentation note is utilised.

The results of this study demonstrate the use of pre-templated operation notes is essential to ensure sufficient documentation of operative procedures. We recommend the use of a pre-templated operation note as part of an electronic, typed record, in accordance with RCSEng guidance.
